# Early Unplanned Reoperation After Glioma Craniotomy: Incidence, Predictor and Process Improvement

**DOI:** 10.3389/fonc.2022.898873

**Published:** 2022-05-06

**Authors:** Yu Zhang, Peigang Ji, Shoujie Wang, Huaizhou Qin, Qing Cai

**Affiliations:** Department of Neurosurgery, Tangdu Hospital, Air Force Military Medical University, Xi’an, China

**Keywords:** early unplanned reoperation, glioma craniotomy, predictor, operating room, quality index

## Abstract

**Objective:**

To evaluate the rate of, reasons for, and predictors of unplanned reoperation after craniotomy for glioma in a single-institution consecutive series.

**Methods:**

Patients who underwent glioma resection at our hospital from 2015 to 2021 were included (n=1563). Multivariate logistic regression was used to examine the predictors of early unplanned cranial reoperation. The predictors that were screened included patient age, sex, tumor properties, blood loss, blood pressure and antiplatelets drugs usage.

**Results:**

A total of 3.6% (56/1563) of the patients underwent an early unplanned reoperation after craniotomy for glioma. The reasons for early unplanned reoperation were brain edema (48.2%), cerebral infarction (33.9%) and hemorrhage (17.9%). The predictors of early unplanned reoperation were WHO grade III-IV, peritumoral edema ≥1 cm, subtotal resection, arterial/venous involvement and elevation in blood pressure ≥50 mmHg.

**Conclusions:**

Glioma properties and blood pressure management are decisive predictors of early unplanned reoperation for glioma resection. The authors provide a nuanced discussion regarding early unplanned reoperations and perioperative process improvement as a quality indicator for glioma patient populations.

## Introduction

Unplanned reoperation (UR) is defined as patients who need unplanned reoperation due to various factors during the same hospitalization (1). Its incidence is currently recognized as an important evaluation index of medical care quality. Unplanned reoperation, especially early unplanned reoperation (EUR, within 7 days), is a negative index of perioperative management because its occurrence may affect the prognosis of patients, prolong the hospitalization time and increase the hospitalization expenses of patients, suggesting the existence of medical deficiencies and hidden medical dangers (2). Therefore, the goal of supervising and analyzing unplanned reoperations is to identify opportunities for technical improvement in the details of the medical process to achieve effective management and ultimately improve the quality of medical care.

Glioma is the most common primary malignant intracranial tumor and accounts for approximately 30% of all primary intracranial tumors (3). The current treatment is comprehensive surgical treatment. Maximum tumor resection and nerve function protection are the keys to a successful operation (4). However, prior studies have shown that the incidence, complications and mortality of EUR for gliomas is the highest after craniotomy of intracranial primary tumors (5, 6). In addition, few studies have evaluated the incidence, risk factors and causes of EUR in glioma patients. We reviewed all return visits to the operating room, over 6 consecutive years, and assessed the following questions: (1) What is the incidence and causes of EUR after craniotomy in patients with glioma? (2) What baseline characteristics and operative variables are independent predictors of EUR in patients with glioma? (3) To understand the mortality and dismal prognosis of EUR at 30 days, optimize the details of perioperative management through a series of measures, reduce the incidence of EUR and improve the quality of surgical treatment.

## Methods

### Patient Selection

We retrospectively analyzed the medical records of 1563 patients with supratentorial gliomas who underwent craniotomy resection between January 2015 and December 2021 at the Department of Neurosurgery, Tangdu Hospital of Air Force Military Medical University (Xi’an, China). Inclusion criteria: Patients with preoperative Karnofsky Performance Scale (KPS) scores >60 and American Society of Anesthesiologists (ASA) grade I-II were followed up for at least 3 months. Exclusion criteria: (1) Emergency surgery; (2) Reoperation of other parts unrelated to the previous operation; (3) History of multiple operations at different stages and times; (4) Diagnostic/therapeutic procedures. All research data were collected in accordance with an active protocol approved by the hospital institutional review board.

### Variables and Data Collection

We identified patients who underwent reoperation within 7 days of the index surgery by reviewing the electronic medical database and the operating room logs. Follow-up data were obtained *via* telephone interviews. Clinical data, such as sex, age, signs and symptoms, and pre- and postoperative CT/MRI findings, was retrieved. Histological type was determined according to WHO grade (WHO grade I-II were low-grade gliomas, WHO grade III-IV were high-grade gliomas). Tumor size: maximum diameters were measured on the T1 + contrast sequences. Extent of peritumoral edema as the maximum axial diameter on gadolinium-enhanced T1-weighted and FLAIR MRI sequences. The tumor location was divided into superficial and deep groups. Extent of resection: gross total resection (GTR) was confirmed on achievement of 100% surgical resection of the preoperative volume of the Gd-enhanced lesion on MRI by using the (A× B × C)/2 method, while subtotal resection (STR) was considered when the resection rate, as measured on postoperative MRI, was < 100% of the preoperative volume, near-total was 90%–99% resection rate and subtotal was 80%–89% resection rate. Arterial/venous involvement: the peritumoral veins or perforating arteries were damaged purposefully or accidentally, according to the imaging findings and intraoperative conditions. Recurrence was defined as the reappearance of the lesion (tumor volume > 25%) after undergoing previous total tumor resection. Postoperative elevated blood pressure was defined as an increase of 50 mmHg in systolic blood pressure upon entering the operating room and lasting for more than half an hour. Postoperative MRI examinations were performed within 48–72 hours after surgery. Clinical outcomes were evaluated using the KPS score before reoperation, at discharge and at 30 days.

### Statistical Analysis

Categorical variables were expressed as numbers (percentages), and the difference was evaluated using the chi-squared test or the Fisher’s exact test, as appropriate. To detect independent risk factors associated with the incidence of unplanned reoperation for glioma, univariate regression analysis was used. Risk factors with a p value < 0.05 in univariate regression analysis were selected for further multivariate regression analysis. Odds ratios (ORs) along with 95% confidence intervals (CIs) were calculated. All statistical analyses were conducted using R software version 4.0 (R Core Team, R Foundation for Statistical Computing, Vienna, Austria; http://www.R-project.org/).

## Results

### Overview of Unplanned Reoperations

Within the study population, a total of 1563 glioma resections were performed during the study period. The clinical data and tumor characteristics of all patients are shown in **Table 1**. The incidence of unplanned reoperations within 7 days was 3.6% (56/1563). The causes of EUR were brain edema, cerebral infarction and hemorrhage. We identified postoperative brain edema, cerebral infarction and hemorrhage as the craniotomy outcomes that were clinically significant and required surgical intervention. The causes of unplanned reoperation were brain edema (48.2%, 27/56), cerebral arterial/venous infarction (33.9%, 19/56) and intracranial hemorrhage (17.9%, 10/56).

**Table 1:** Baseline characteristics of the patients.

Variable	Overall	Non-reoperations	Reoperations	*P*
	(n = 1563)	(n = 1507)	(n = 56)	
Gender				0.080
Female	637 (40.8%)	621 (41.2%)	16 (28.6%)	
Male	926 (59.2%)	886 (58.8%)	40 (71.4%)	
Age (years)				0.196
<60	618 (39.5%)	601 (39.9%)	17 (30.4%)	
≥60	945 (60.5%)	906 (60.1%)	39 (69.6%)	
WHO grade				<0.001
I-II	558 (35.7%)	551 (36.6%)	7 (12.5%)	
III-IV	1005 (64.3%)	956 (63.4%)	49 (87.5%)	
Tumour size (mm)				0.087
<40	574 (36.7%)	560 (37.2%)	14 (25.0%)	
≥40	989 (63.3%)	947 (62.8%)	42 (75.0%)	
Tumor localization				0.096
Superficial	1147 (73.4%)	1100 (73.0%)	47 (83.9%)	
Deep	416 (26.6%)	407 (27.0%)	9 (16.1%)	
Peritumoral edema				0.001
<1 cm	989 (63.3%)	966 (64.1%)	23 (41.1%)	
≥1 cm	574 (36.7%)	541 (35.9%)	33 (58.9%)	
Extent of resection				0.004
Gross total/near total	921 (58.9%)	899 (59.7%)	22 (39.3%)	
Subtotal	642 (41.1%)	608 (40.3%)	34 (60.7%)	
Arterial/venous involvement				0.024
No	720 (46.1%)	703 (46.6%)	17 (30.4%)	
Yes	843 (53.9%)	804 (53.4%)	39 (69.6%)	
Recurrent				0.602
No	1081 (69.2%)	1040 (69.0%)	41 (73.2%)	
Yes	482 (30.8%)	467 (31.0%)	15 (26.8%)	
Blood loss (mL)				0.384
<600	773 (49.5%)	749 (49.7%)	24 (42.9%)	
≥600	790 (50.5%)	758 (50.3%)	32 (57.1%)	
Elevated BP (mmHg)				0.033
<50	900 (57.6%)	876 (58.1%)	24 (42.9%)	
≥50	663 (42.4%)	631 (41.9%)	32 (57.1%)	
Antiplatelet drugs usage				0.107
No	1317 (84.3%)	1265 (83.9%)	52 (92.9%)	
Yes	246 (15.7%)	242 (16.1%)	4 (7.1%)	

WHO, World Health Organization; BP, blood pressure.

Among them, there were 7 cases of edema, infarction and hemorrhage with hydrocephalus, The hydrocephalus was relieved after the hematoma evacuations or the decompressive craniectomy. The surgical procedures described as unplanned reoperation included decompressive craniectomy (46/56), hematoma evacuations (7/56), hematoma evacuations + decompressive craniectomy (3/56). The indication for reoperation was determined by the attending neurosurgeon based on the patient’s clinical course and investigations. Reoperation occurred in 3 patients within 6 hours, in 2 patients within 12 hours, in 5 patients within 24 hours, in 19 patients within 48 hours, and in 18 patients within 72 hours. Nine patients underwent reoperation between Day 4 and Day 7 after surgery.

Timely and effective reoperation can significantly improve the patient’s prognosis. Of the 56 patients who underwent reoperation, 31 were normal or asymptomatic, 17 practiced self-care, and only 5 needed special care after reoperation at 30 days. KPS score significantly changed before reoperation, at discharge and at 30 days. Surgical mortality, defined as death within 30 days of reoperation, was 0.32% (5/1563). The cause of death in 2 patients was postoperative edema/infarction and subsequent herniation, postoperative infection in 2 patients, and seizure in 1 patient (**Table 2**).

**Table 2:** Clinical outcomes of unplanned reoperation after glioma surgery.

Parameter	Reop preoperative	Reop at discharge	Reop at 30-days
**KPS scale**			
** ≥ 90**	0	21 (37.5%)	31 (55.3%)
** 60–80**	24 (42.9%)	23 (41.1%)	17 (30.4%)
** ≤ 50**	32 (57.1%)	10 (17.8%)	5 (8.9%)
**Death**	0	2 (3.6%)	3 (5.4%)

Reop, reoperation; KPS, Karnofsky Performance Scale.

### Predictors of Reoperation

#### Univariate Analysis

Patients with a WHO grade III-IV had an unplanned reoperation significantly more often than those with a WHO grade I-II (87.5% vs. 12.5%, p <0.001, OR 3.95, 95% CI 1.89-9.71). Peritumoral edema ≥1 cm was significantly more often associated with unplanned reoperation than peritumoral edema <1 cm (58.9% vs. 41.1%, p =0.001, OR 2.55, 95% CI 1.49-4.46). Patients undergoing subtotal resection had an unplanned reoperation significantly more often than those undergoing gross/near total resection (60.7% vs. 39.3%, p= 0.004, OR 2.28, 95% CI 1.33-4.00). Patients with arterial/venous involvement had an unplanned reoperation significantly more often than those without arterial/venous involvement (69.6% vs. 30.4%, p = 0.024, OR 1.99, 95% CI 1.13-3.66). Patients with an elevated BP ≥50 mmHg had an unplanned reoperation significantly more often than those with an elevated BP <50 mmHg (57.1% vs. 42.9%, p = 0.033, OR 1.85, 95% CI 1.08-3.20) (**Table 3**).

**Table 3:** Clinical risk factors for prediction of reoperations after glioma resection.

Variable	Univariable analysis		Multivariable analysis	
	*OR (95%CI)*	*P*	*OR (95%CI)*	*P*
Gender (Male vs Female)	1.74 (0.98-3.24)	0.080		
Age (years) (≥60 vs <60)	1.51 (0.86-2.78)	0.196		
WHO grade (III-IV vs I-II)	3.95 (1.89-9.71)	<0.001	3.96 (1.77-8.84)	<0.001
Tumour size (mm) (≥40 vs <40)	1.76 (0.97-3.38)	0.087		
Tumor localization (Deep vs Superficial)	0.53 (0.24-1.03)	0.096		
Peritumoural edema (cm) (≥1 vs <1)	2.55 (1.49-4.46)	0.001	2.39 (1.38-4.15)	0.002
Extent of resection (Subtotal vs Gross/near total)	2.28 (1.33-4.00)	0.004	2.18 (1.25-3.79)	0.006
Arterial/venous involvement (Yes vs No)	1.99 (1.13-3.66)	0.024	1.91 (1.06-3.43)	0.031
Recurrent glioma (Yes vs No)	0.82 (0.43-1.47)	0.602		
Blood loss (mL) (≥600 vs <600)	1.32 (0.77-2.28)	0.384		
Elevated BP (mmHg) (≥50 vs <50)	1.85 (1.08-3.20)	0.033	1.86 (1.07-3.22)	0.026
Antiplatelets drugs usage (Yes vs No)	0.40 (0.14-1.12)	0.082		

WHO, World Health Organization; BP, blood pressure.

#### Multivariate Analysis

We performed a multivariate logistic regression analysis to identify potential predictors of postoperative unplanned reoperation in glioma patients. WHO grade (III-IV) (p<0.001, OR 3.96, 95% CI 1.77-8.84), peritumoral edema ≥1 cm (p=0.002, OR 2.39, 95% CI 1.38-4.15), subtotal resection (p=0.006, OR 2.18, 95% CI 1.25-3.79), arterial/venous involvement (p = 0.031, OR 1.91, 95% CI 1.06-3.43) and elevated BP ≥50 mmHg (p = 0.026, OR 1.86, 95% CI 1.07-3.22) were identified as independent and significant predictors of postoperative venous infarction (**Table 3**).

## Discussion

Previous research from the neurosurgical literature has suggested that reoperation may be a useful quality indicator, and few studies have examined reoperation and death after glioma craniotomy. McLaughlin (7) evaluated early reoperation (within 7 days) among all neurosurgical procedures (cranial and spine) at a single institution, reporting a 2.6% reoperation rate. Dasenbrock (5) analyzed the rates, causes, and predictors of unplanned cranial reoperation after craniotomy for tumors. The 30-day reoperation rate was 3.1%, and mortality was 3.2%. Lassen (6) reviewed reoperation after craniotomy for tumors at 2 institutions. The 30-day mortality was 2.3%, and the mortality rate for high gliomas was 2.9%.

In the single-center data analysis, 1563 glioma patients were analyzed; the early unplanned cranial reoperation rate was 3.6%, and the 30-day mortality was 0.32%. The reported population (5–7) included patients with acute and critical intracranial tumors and metastases that underwent neurosurgery. However, the patients we included had single supratentorial gliomas, underwent elective surgery and had a good preoperative evaluation. Therefore, the mortality was significantly lower than that of the reported population, but the reoperation rate was higher. A higher reoperation rate was strongly associated with glioma risk factors.

### Causes and Risk Factors of Early Unplanned Reoperation

**Figure 1:**
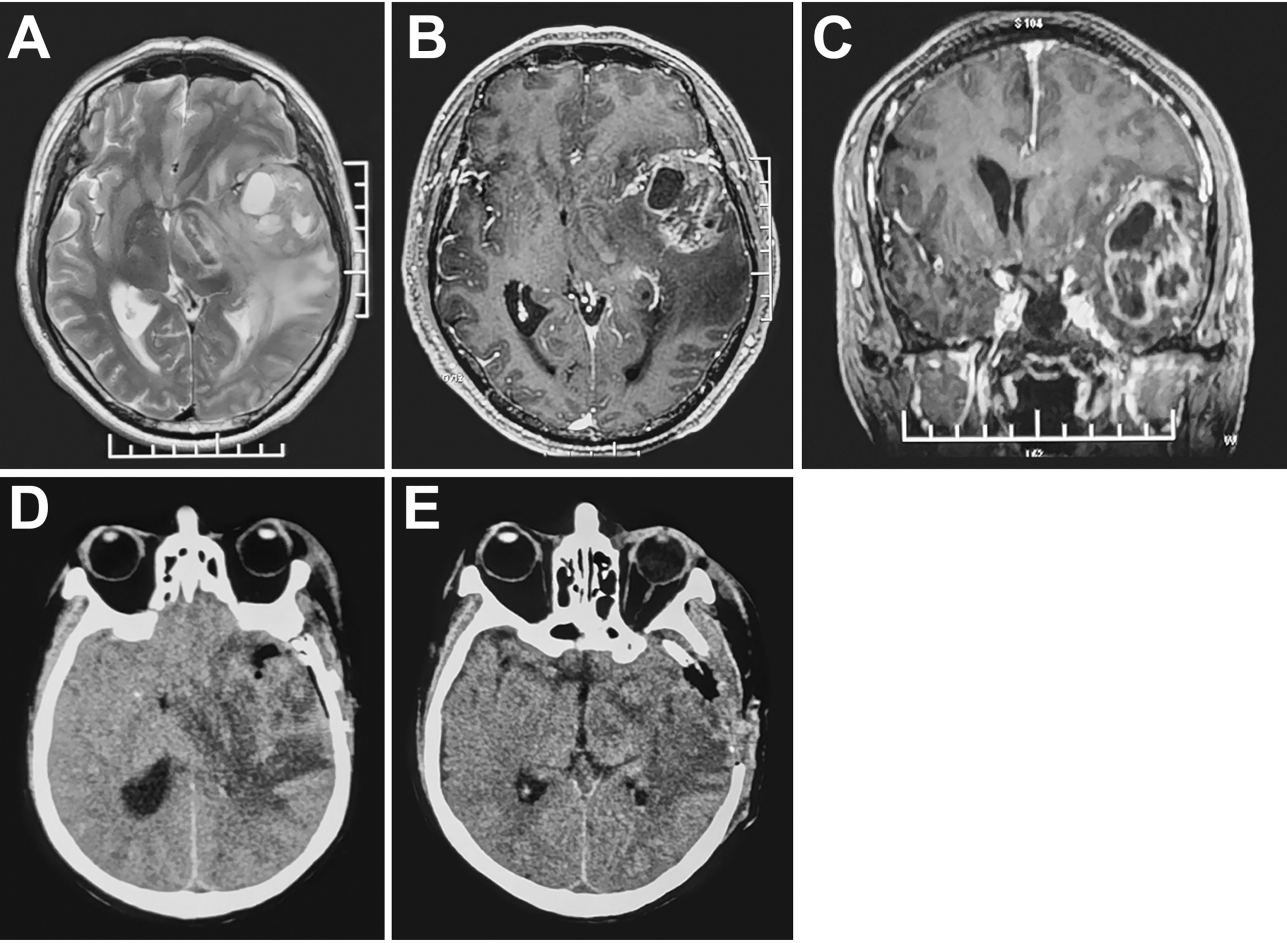
MRI **(A-C)** high-grade glioma with marked peritumoral edema in the left temporal lobe. CT **(D)** subtotal tumor resection, obvious midline shift and increased peritumoral edema 48 hours postoperatively. CT **(E)** Resection of residual tumor and decompressive craniectomy.

**Figure 2:**
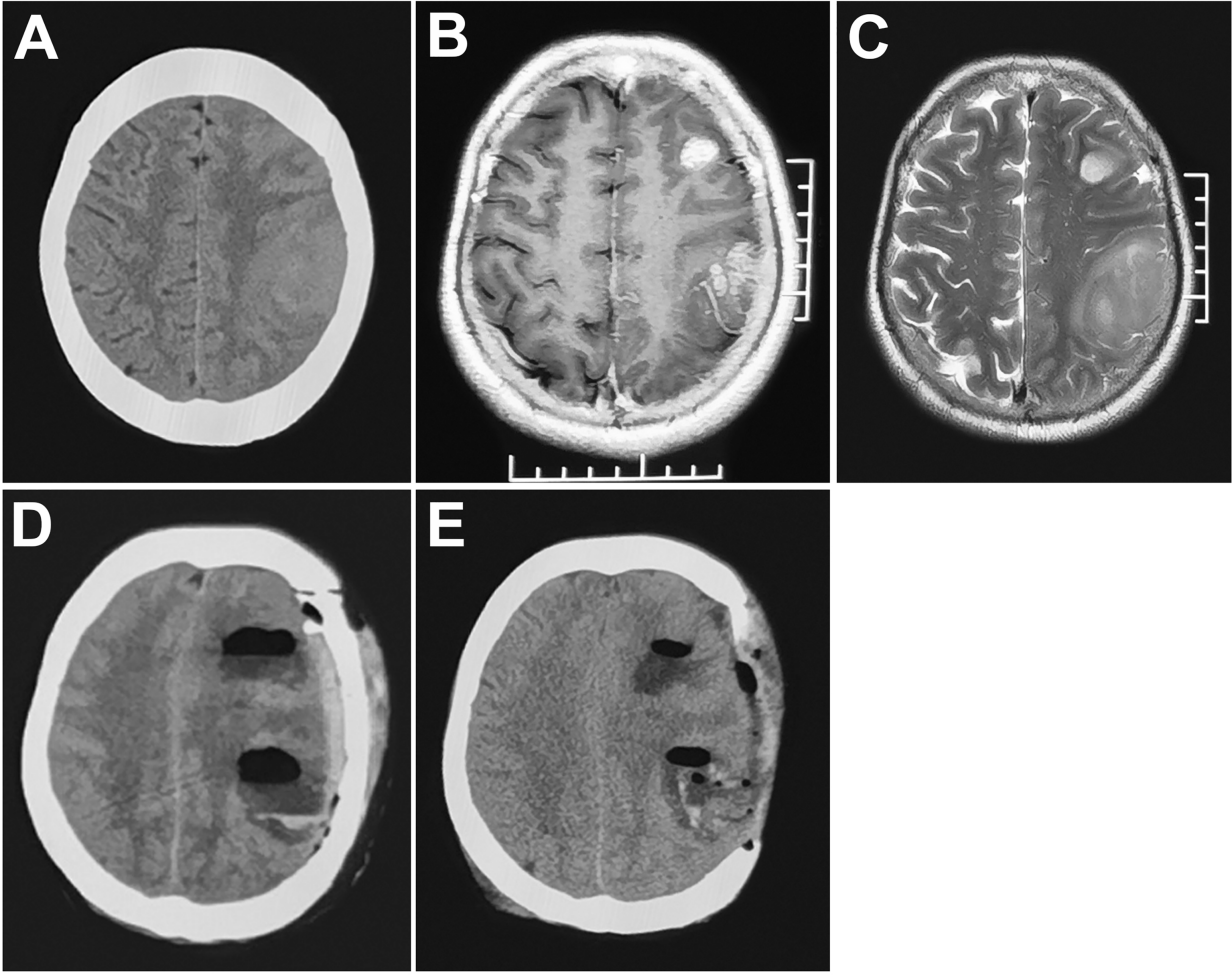
MRI **(A–C)** multiple gliomas in the left frontoparietal lobe. CT **(D)** obvious midline shift and cerebral venous infarction (cortical veins were injured during the operation) 72 hours postoperatively. **(E)** decompressive craniectomy.

EUR was performed for elevated intracranial pressure caused by edema, infarction and hemorrhage. Symptoms and signs, such as consciousness, pupillary reflex, vital signs and neurological function, were observed after the operation. Combined with urgent cranial CT, the following conditions can be considered for reoperation (**Figures 1**, **2**): 1. Progressive decline of consciousness and neurological function 2. Acute cerebral hernia formation. The causes of progressive decline in consciousness, neurological dysfunction and formation of cerebral hernia are directly related to the imaging manifestations (infarction, edema and hemorrhage in the operation area), showing an upward trend in intracranial pressure (midline deviation, disappearance of cistern).

Related studies showed that the rate of reoperation for hemorrhage after craniotomy was 2.1% (6) and 1.5% (8), respectively, and edema/infarction was 0.3% (7). Our postoperative hemorrhage rate was 0.6%, and edema/infarction was 2.9%. Unlike previous unplanned reoperations in neurosurgery, the hemorrhage rate of unplanned reoperation in our case series was significantly lower than that reported in the literature, but the edema/infarction rate was obviously higher than that reported in the literature, which may be related to the nature of the glioma. Several factors contribute to intracranial edema, infarction and hemorrhage following glioma resection, and independent predictors of reoperation in this analysis were WHO grade (III-IV), peritumoral edema ≥1 cm, subtotal resection, arterial/venous involvement and elevated BP ≥50 mmHg.

The potential causes of EUR may be local or systemic. Locally, partial resection of high-grade gliomas causes postoperative bleeding, and tumor vessels lacking normal architecture or vessels in residual tumors cause capillary rebleeding during blood pressure increases (9, 10). In addition, brain tissue traction during tumor resection, malignant cerebral edema with residual tumor cytotoxicity and cerebral ischemic infarction after arteriovenous vascular injury aggravated the edema/infarction of the original brain tissue and caused a rapid increase in intracranial pressure around the tumor after surgery, leading to early reoperation. Systemic factors include fluctuations in systolic blood pressure. A study suggested that intraoperative and postoperative systolic blood pressure >160 mmHg was associated with postoperative craniotomy hematoma requiring evacuation (11). This was basically consistent with our conclusion that systolic blood pressure was increased by 50 mmHg in the operating room.

Although our results suggested that intraoperative blood loss and advanced age were not high-risk factors for postoperative bleeding, many reports showed that they are potential bleeding factors. A median blood loss of 500 mL (12) increases the rate of postoperative hemorrhage because of reduced platelets and coagulation factors. Age >60 (13) was significantly correlated with an increased risk of postoperative hematomas and has been attributed to the tissue fragility observed in elderly patients. Massive hemorrhage leads to a hypocoagulable state, and advanced age reduces the compliance of brain tissue or blood vessels, which can cause hemorrhage in the surgical, epidural or subdural areas. Small hematomas can also stimulate compression of brain tissue, secondary to vasospasm or occlusion, exacerbating the degree of edema and infarction. The long-term use of antiplatelet drugs was an important factor that caused difficulty in intraoperative hemostasis and postoperative bleeding. Through preoperative timely withdrawal of drugs, we can correct the patient’s physiological function as much as possible before the operation to ensure the safety of the operation. Therefore, anticoagulation in elective surgery was not a risk factor for EUR.

### Perioperative Process Improvement of the Index Surgery

Perioperative prevention of risk factors reduces the incidence of EUR and mortality after craniotomy for patients with glioma. Control of underlying diseases, such as hypertension, diabetes, renal function, coagulation function, etc., adjusted the patient’s preoperative comprehensive index (ASA and KPS) to the greatest extent. Intraoperative neuronavigation, neurophysiological monitoring and intraoperative wake-up techniques are used to maximize tumor resection (14). Gentle surgical operation avoids damage to peritumoral arteries and venous vessels and purposefully increases blood pressure to 140-150 mmHg after tumor resection and hemostasis. Evaluate the benefit of a “5-minute bleeding time-out” by waiting and assessing hemostasis in “oozy” patients (7). Standardize the use of tack-up sutures. Postoperative blood pressure management: strict anesthesiologic protocol to achieve blood pressure management (15): ① A deep opioid analgesia to eliminate any acute elevations of the arterial pressure during and immediately after craniotomy. ② Emergence from anesthesia was delayed for an average of 1.5 to 2 hours following the neurosurgical procedure. ③A 24-48-hour observation period in the recovery area/intensive care unit after craniotomy before transfer to a neurosurgical ward for further nursing care should be sufficient for most patients (16).

## Conclusion

A comprehensive analysis of reoperations in patients with glioma characteristics describes and identifies the incidence, operation time, causes, and risk factors of EURs. By combining perioperative glioma characteristics, intraoperative monitoring technology, and multidisciplinary management (anesthesia and intensive care), the rate of early unplanned reoperations can be reduced. Only with a comprehensive understanding of all aspects of glioma reoperation can a quality improvement program be strategically initiated to successfully reduce reoperation rates and improve the quality of delivered care.

## Data Availability Statement

The original contributions presented in the study are included in the article/supplementary material. Further inquiries can be directed to the corresponding authors.

## Author Contributions

YZ: Conceptualization, Methodology, Writing – original draft. PJ: Writing - original draft. SW: Writing - original draft. HQ: Supervision. QC: Writing - review and editing. All authors contributed to the article and approved the submitted version.

## Conflict of Interest

The authors declare that the research was conducted in the absence of any commercial or financial relationships that could be construed as a potential conflict of interest.

## Publisher’s Note

All claims expressed in this article are solely those of the authors and do not necessarily represent those of their affiliated organizations, or those of the publisher, the editors and the reviewers. Any product that may be evaluated in this article, or claim that may be made by its manufacturer, is not guaranteed or endorsed by the publisher.

## Abbreviations

UR, unplanned reoperation; EUR, early unplanned reoperation; KPS, karnofsky performance scale; ASA, American society of anesthesiologists; WHO, World Health Organization.
